# A Study on the Assembly Mechanisms of Shrub Communities in Coniferous and Broadleaved Forests—A Case Study of Jiangxi, China

**DOI:** 10.3390/biology14121683

**Published:** 2025-11-26

**Authors:** Yuxi Xue, Xiaoyue Guo, Wei Huang, Xiaohui Zhang, Yuxin Zhang, Yongxin Zhong, Xia Lin, Qi Zhang, Qitao Su, Yian Xiao

**Affiliations:** Key Laboratory of Jiangxi Province for Biological Invasion and Biosecurity, School of Life Sciences, Jinggangshan University, Ji‘an 343009, China; xueyuxi1@jgsu.edu.cn (Y.X.); guoxiaoyue@jgsu.edu.cn (X.G.); 2408301021@jgsu.edu.cn (W.H.); zhangxiaohui@jgsu.edu.cn (X.Z.); 2308301009@jgsu.edu.cn (Y.Z.); zhongyongxin@jgsu.edu.cn (Y.Z.); linxia@jgsu.edu.cn (X.L.); 2408301007@jgsu.edu.cn (Q.Z.)

**Keywords:** community assembly, functional diversity, functional traits, understory shrubs, soil nitrogen driving

## Abstract

The ecological strategies of understory shrubs are critical for maintaining the structure and function of forest understory vegetation. Understanding the assembly mechanisms of these shrub communities is a central issue in modern ecology. To address this, our study was conducted in the typical red soil regions of Jiangxi, China, focusing on secondary forests (including both broadleaved and coniferous types) of similar stand age. We aimed to assess the effects of various environmental factors—such as soil pH, total nitrogen content, bulk density, and understory temperature—along with tree-layer characteristics—including canopy closure, tree species richness, and diameter at breast height (DBH)—on the species composition, functional traits, and phylogenetic structure of the shrub layer. Results showed: One-way ANOVA revealed significant differences in functional traits between the two forest types. Specifically, leaf thickness, specific leaf area, and chlorophyll content were significantly higher in the coniferous forest, whereas leaf dry matter content was significantly lower compared to the broadleaved forest (*p* < 0.05). These results suggest that understory shrubs in the coniferous forest primarily adopt a resource-conservative strategy, while those in the broadleaved forest exhibit a resource-acquisitive strategy. Phylogenetic analysis further revealed that the phylogenetic diversity (PD) of coniferous forests was significantly lower than that of broadleaved forests (*p* < 0.05). The phylogenetic structure in coniferous forests showed a more clustered pattern (NTI > 0, NRI > 0), suggesting stronger environmental filtering. Diversity index analysis showed that the Chao1 index indicated a richer potential species pool in broadleaved forests (*p* < 0.05), while species distribution was more even in coniferous forests (*p* < 0.05). Random Forest model analysis identified the diameter at breast height (DBH) of trees as the most critical negative driver, while soil pH was the primary positive driver. Redundancy Analysis (RDA) confirmed that the community structure in coniferous forests was mainly driven by biotic competition pressure represented by DBH, whereas the structure in broadleaved forests was more closely associated with abiotic factors like soil total nitrogen and pH (R^2^ = 0.29, *p* < 0.05). These environmental drivers, through strong environmental filtering, collectively resulted in a phylogenetically clustered pattern of shrub communities in both forest types. This study demonstrates that the assembly of understory shrub communities is a complex, multi-level process co-regulated by multiple factors, shaped by both the biotic pressure from the overstory structure and abiotic filtering from the soil environment. This finding deepens our understanding of the rules governing community assembly in forest ecosystems.

## Simple Summary

The assembly of understory shrub communities is a fundamental ecological process, yet the synergistic mechanisms driven by overstory trees and soil properties remain inadequately quantified. This study investigated the composition, functional traits, and phylogenetic structure of shrub layers in subtropical coniferous and broadleaved forests. Findings reveal a divergence in ecological strategies: shrub species in coniferous forests exhibited functional traits associated with a resource-conservative strategy (e.g., higher leaf thickness and chlorophyll content), whereas those in broadleaved forests displayed characteristics indicative of a resource-acquisitive strategy. Phylogenetic analyses indicated significantly lower phylogenetic diversity and a more clustered structure in coniferous forests, reflecting stronger environmental filtering. Statistical models identified tree diameter at breast height (DBH) as the primary negative driver and soil pH as the main positive driver of community assembly. Overall, these results demonstrate that the assembly of understory shrubs is a multi-level process co-regulated by biotic pressure from the overstory and abiotic filtering from the soil environment, providing novel insights into the maintenance of biodiversity in forest ecosystems.

## 1. Introduction

The structure and function of forest ecosystems form the foundation for maintaining biodiversity and ecosystem services. As a crucial component of forests, understory shrubs play a critical role in sustaining below-canopy ecological processes. Moreover, understory vegetation contributes significantly to species diversity, nutrient cycling, microenvironment regulation, and the facilitation of tree regeneration [1]. Although the shrub layer itself has limited productivity, its diversity is as crucial in sustaining the overall ecosystem functioning of forests [2]. Investigating the assembly mechanisms of understory plant communities is of profound significance for understanding the successional dynamics of forest ecosystems and enhancing their multi-functionality. Plant functional traits serve as direct indicators of ecological processes in community assembly by characterizing the fundamental trade-offs between resource acquisition and conservation strategies [3]. By comparing leaf functional traits across different forest types, Wang L et al. analyzed the divergence in ecological strategies among three forest types. Their findings demonstrated a trade-off within the leaf economics spectrum between deciduous and evergreen subcommunities, reflecting distinct survival strategies linked to differential resource utilization. This ultimately underscores a trade-off associated with the differentiation of leaf functional traits [4]. Functional diversity, as a multidimensional aspect of biodiversity, quantifies the differences and spatial distribution of functional traits among plants within ecosystems. This trait-based approach offers a more direct characterization of plant–environment interactions, thereby yielding deeper insights into species composition, niche partitioning, and interspecific relationships within plant communities [5]. Moreover, plant functional traits play critical roles in multiple ecosystem processes, directly influencing ecosystem functions and services [6], and thus helping in clarify the formation and structure of plant functional communities.

In forest ecosystems, the Specific leaf area (SLA) and Leaf dry matter content (LDMC) of understory shrubs represent a central trade-off along the “acquisition–conservation” spectrum. Shrubs with high SLA—characterized by a larger leaf area per unit dry mass—typically exhibit rapid light acquisition and photosynthetic rates, supporting faster growth in resource-rich environments, at the expense of structural durability and herbivore resistance [7]. In contrast, shrubs with high LDMC enhance tissue density and defensive capacity, improving survival in resource-limited settings such as nutrient-poor soils or waterlogged swamps, but usually at the cost of reduced light capture efficiency and growth rate [8]. This functional dichotomy is further reflected in growth forms: deciduous shrubs generally possess higher SLA and lower LDMC, aligning with a resource-acquisitive strategy, whereas evergreen shrubs tend to exhibit higher LDMC and lower SLA, consistent with a long-term resource conservation strategy [9]. Overall, the variation in these key functional traits is driven by environmental gradients—including light availability, moisture, and soil pH—while also being influenced by phylogenetic constraints and functional group identity [10].

Functional diversity of shrubs quantifies the divergence, range, and distribution of functional traits among species within a community, directly reflecting their strategies for environmental adaptation and interspecific interactions. It provides deeper mechanistic insights into the linkages between community assembly processes and ecosystem functioning than traditional species diversity metrics [5]. Under external environmental stress, indices such as Functional Richness Indices (Fric) and Functional Dispersion Indices (FDis) exhibit higher sensitivity in detecting community responses. For instance, marked differences in functional diversity have been documented among woody plants of different growth forms, reflecting distinct ecological strategies adopted to cope with environmental constraints and enhance competitive ability [11]. Complementarily, phylogenetic community ecology infers assembly mechanisms by analyzing the evolutionary relationships among species. The Net Relatedness Index (NRI), a key metric in this field, quantifies the deviation of the observed phylogenetic relatedness of co-occurring species from a null expectation based on the regional species pool. This enables researchers to discern whether environmental filtering or competitive exclusion predominantly structures a community. Understory communities (including shrubs and herbs) follows predictable shifts with stand development. Furthermore, altitudinal gradients significantly influence phylogenetic relatedness patterns [12]. Specifically, communities at lower elevations are primarily structured by competitive exclusion, while those at higher elevations appear to be maintained by a combination of habitat filtering and competitive processes [13].

Soil environmental conditions have been established as primary drivers of ecosystem multifunctionality [14]. Research by Sanaei and Anvar indicates that soil bacterial and fungal diversity correlate more strongly with the diversity and composition of tree functional traits than with tree species richness. This linkage between tree and soil microbial diversity is mediated by soil stoichiometry [15]. Soil pH represents another critical abiotic factor influencing ecosystem multifunctionality [16,17]. Its effects on diversity vary across spatial contexts and communities. For instance, Merunková’s study demonstrated that soil pH enhances plant richness in grassland ecosystems [18]. However, some studies report non-significant effects of pH on biodiversity [19], while others document hump-shaped distributions of plant diversity along soil pH gradients [20]. Beyond pH, soil nitrogen content significantly shapes biological communities. Nitrogen not only directly affects plants but also indirectly influences plant communities by altering soil microbial dynamics [21]. Prober and Wiehl’s research identified total nitrogen as the nutrient most strongly associated with plant diversity [22]. Elevated soil phosphorus and nitrogen levels can mitigate plant abiotic stress, enhance pathogen resistance, and ultimately modify the composition and diversity of plant communities [23].

Community assembly mechanisms operate across multiple levels and are influenced by various environmental factors. Research indicates that water availability is a primary driver of compositional shifts in forests and savannas [24]. For instance, Yao et al. found that plant diversity in herbaceous and woody communities within China’s arid regions is primarily regulated by soil nutrients and precipitation, respectively [25]. However, environmental factors are not always the dominant drivers; studies in some habitats suggest that stochastic processes can prevail. For example, Angulo’s research on dune plant communities revealed that random assembly appears to be the principal factor structuring the studied communities, with environmental filtering potentially being a secondary contributor to the observed phylogenetic structure. This suggests that both deterministic and stochastic processes can operate simultaneously to mediate the assembly of dune plant communities [26]. Furthermore, community assembly is not static but varies across both temporal and spatial scales. The relative importance of different processes can shift from early to late successional stages and is influenced by the observational scale [27,28].

As a key component of the forest understory, shrubs are both a product of environmental filtering and a reflection of competitive interactions. They face a complex microenvironment [29], characterized by canopy shading, reduced diurnal temperature fluctuations, and decreased soil water recharge due to rainfall interception by the tree canopy [30,31,32]. As fundamental components of the forest understory, shrubs perform ecological roles that are as critical as those of the tree canopy. Nevertheless, the mechanisms driving the assembly of subtropical understory shrub communities are still not well elucidated. A clearer understanding of these assembly mechanisms is therefore crucial, as it will not only enhance our ability to sustainably manage and utilize forest ecosystem services but also provide fundamental insights into the structure and functioning of forest communities. This study aims to address the following questions: 1. Do the functional traits of understory shrubs differ between the two forest types, and what are the patterns of functional trait divergence? 2. If differences exist, what are the primary drivers responsible for the divergent assembly of the understory shrub communities?

## 2. Materials and Methods

### 2.1. Research Plots

**Figure 1:**
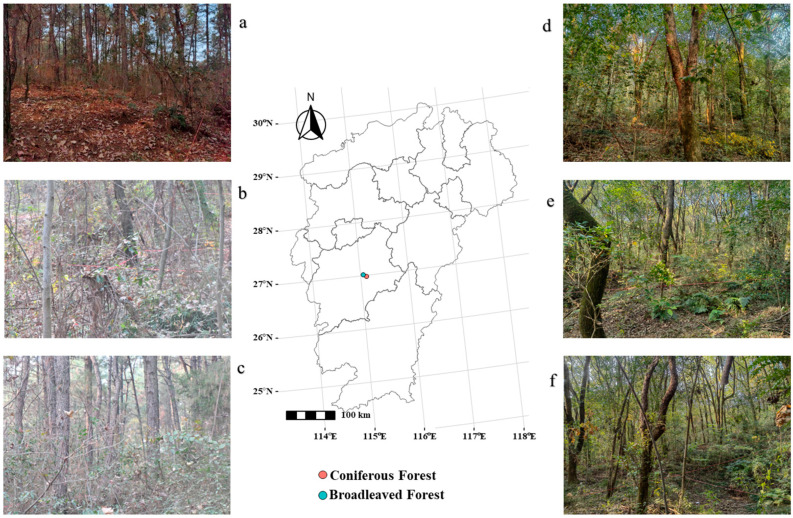
Overview of the study area. Note: (**a**–**c**) are understory shrubs in some sample plots of coniferous forests; (**d**–**f**) are understory shrubs in some sample of broadleaf forests.

Ji’an City (25°58′32″ N–27°57′50″ N, 113°46′ E–115°56′ E) is situated in the central part of Jiangxi Province, China, characterized by a humid subtropical climate with distinct seasons, mild and humid conditions, ample sunlight, and abundant precipitation. The average annual temperature is approximately 18.3 °C, with an average annual rainfall of 1457.5 mm. The region experiences a prolonged frost-free period and an annual temperature range of about 40 °C. The dominant soil type across Jiangxi Province is ferrallitic soil (red soil), with the primary vegetation cover consisting of evergreen broad-leaved forests, deciduous broad-leaved forests, and coniferous forests (Figure 1).

Through field surveys and consultations with local communities, two forest stands with comparable ages (approximately 40 years) were selected as study sites. Both plots are secondary forests that have developed through natural succession following the planting of seedlings after the original forest farm was cleared. They now receive government protection. To minimize edge effects, six replicate quadrats were established for each forest type, with a minimum spacing of 100 m between adjacent quadrats. All quadrats were demarcated using PVC pipes to ensure consistent sampling areas. Environmental factors of the sampling plots are summarized in Table 1.

In the investigation of tree layer factors, we surveyed all trees with a diameter at breast height (DBH) greater than 5 cm. For each tree, we measured its DBH and identified the species. The scientific names of the species were then verified and corrected by referring to the Flora of China. Furthermore, we calculated various diversity indices of the tree layer, including species diversity, richness, and evenness, as detailed in Table 2.

#### 2.1.1. Phylogenetic Diversity of Understory Plants

Species within the established quadrats were initially identified in the field. Species names were verified and corrected by referring to the Flora of China (http://www.iplant.cn/ accessed on 25 October 2025). A total of 40 plant species recorded across the 12 quadrats were used as the species pool. The family, genus, and species information for all angiosperms were systematically organized according to the Angiosperm Phylogeny Group III classification system (APG III) [33]. A phylogenetic tree for the understory shrub layer plants in the eucalyptus plantation was subsequently constructed using R 4.4.1 software. All phylogenetic analyses were performed with the picantepackage in R [34].

#### 2.1.2. Functional Trait Analysis

For each species, three healthy and fully expanded leaves were randomly sampled from individuals within the quadrats to ensure three biological replicates. The following leaf functional traits were measured: leaf length, width, and thickness were measured using a vernier caliper; leaf area was determined from leaf images analyzed with ImageJ software (v1.8.0.345). The sampled leaves were transported to the laboratory, where their fresh weight was immediately measured using an electronic balance. Subsequently, the leaves were oven-dried at 70 °C until a constant dry weight was achieved. The leaf chlorophyll content was measured using a plant nutrient meter (Model: JC-YLS; accuracy: ±1.0 SPAD).

#### 2.1.3. Shrub Diversity and Environmental Factor Survey

Shrub species within the quadrats were identified through direct observation. For specimens that could not be confidently identified in the field, voucher samples were collected and identified by consulting specialists and cross-referencing with the Flora of China database to confirm their scientific names. This survey recorded data on shrub and tree species diversity and phylogenetic diversity. Concurrently, several environmental variables were measured: soil bulk density was determined using a 100 cm^3^ soil core sampler; understory temperature was monitored with a TMS (Soil Measurement System) data logger; canopy closure (canopy density) was quantified using a fisheye lens photographic method. Soil samples were collected from four points within each plot using a four-point sampling method. These samples were air-dried in the shade before analysis. Soil pH was measured potentiometrically, and total soil nitrogen content was determined using a fully automated Kjeldahl nitrogen analyzer.

### 2.2. Data Analysis

Data were initially pre-processed using Microsoft Excel 2019. All statistical analyses and visualizations were performed in R version 4.4.1. Data visualization was primarily conducted using the ggplot2 package (v4.0.1) [35]. Weighted functional diversity indices were computed with the FD and ade4 packages (v1.7.23) [36,37]. The phylogenetic tree was generated using the V.PhyloMaker 2 package (v0.1.0) [38]. Differences in shrub functional diversity, phylogenetic diversity, and species diversity (e.g., Shannon-Wiener index, Simpson index) between the two forest types were assessed using the non-parametric Wilcoxon rank–sum test.

To reduce the dimensionality of the data while preserving its integrity, Principal Component Analysis (PCA) was applied separately to three modules: functional diversity, species diversity, and phylogenetic diversity of the shrubs. If the first principal component (PC1) explained more than 70% of the total variance, it was used as a surrogate for the original module matrix. If PC1 explained less than 70% of the variance, a weighted combination of the first and second principal components (PC1 and PC2) was used instead. To investigate the integrated influence of environmental factors and tree layer characteristics on the overall shrub community, Redundancy Analysis (RDA) was performed using the “vegan” package (v 2.6.10) to identify the primary factors driving shrub community assembly [39].

## 3. Results

### 3.1. Analysis of Shrub Survey and Leaf Functional Trait Differences

The survey documented a total of 40 shrub species, representing 27 families and 24 genera. Analysis revealed distinct patterns in the relative abundance of shrub families between the two forest types. Species from the Aquifoliaceae and Smilacaceae families showed higher relative abundance in the coniferous forest (Table 3). In contrast, the Rosaceae, Rubiaceae, and Moraceae families were more abundant in the broadleaved forest. Furthermore, the distribution of relative abundance among shrub species demonstrated a clear divergence between forest types: shrub abundance was more evenly distributed across species in the broadleaved forest, while it was more concentrated in the coniferous forest, indicating higher species dominance in the latter ecosystem.

**Figure 2:**
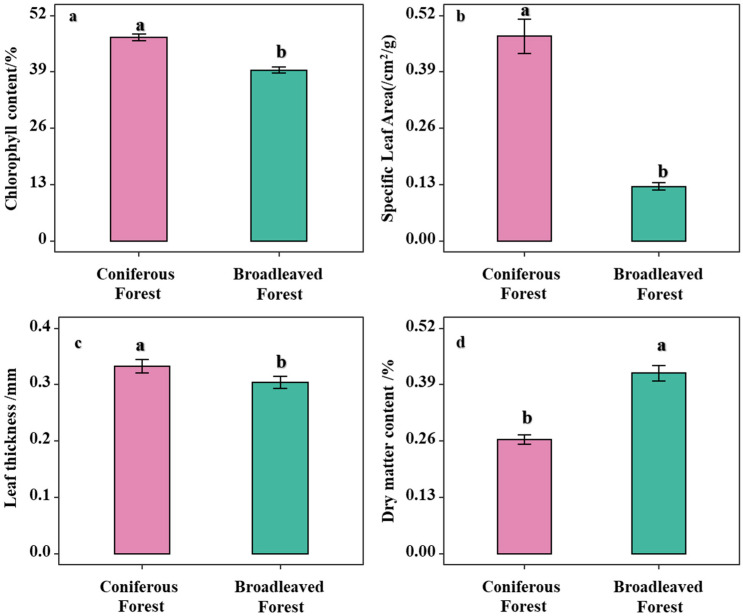
Comparison of functional diversity between Coniferous Forest and Broadleaved Forest. Note: Different letters indicate significant differences in the one-way analysis of variance (one-way ANOVA). (**a**) Analysis of leaf chlorophyll content across different forest types; (**b**) Analysis of SLA of shrubs across different forest types; (**c**) Analysis of leaf thickness of shrubs across different forest types; (**d**) Analysis of LDMC of shrubs across different forest types.

Analysis of the differences in leaf functional traits of understory shrubs between broadleaved and coniferous forests revealed that leaf thickness, specific leaf area (SLA), and chlorophyll content were significantly higher in shrubs under the two coniferous forests compared to those in the broadleaved forest (*p* < 0.05; Figure 2a–c). In contrast, leaf dry matter content (LDMC) was significantly lower in the broadleaved forest (Figure 2d). These results indicate that the coniferous forest environment acts as a strong environmental filter, selecting for shrubs with a specific resource-conservative strategy, whereas the broadleaved forest environment allows for the coexistence of a greater diversity of shrubs employing a resource-acquisitive strategy.

### 3.2. Analysis of Differences in Leaf Functional Diversity Between Two Forest Types

**Figure 3:**
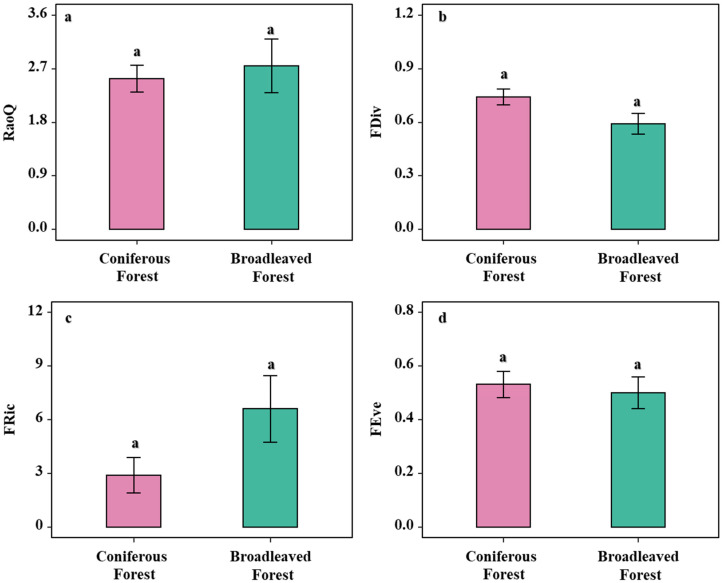
Analysis of shrub functional diversity under two forest types. (**a**) Analysis of differences in shrub Rao’s Q index across forest types; (**b**) Analysis of differences in shrub FDiv index across forest types. (**c**) Analysis of differences in shrub FRic across forest types; (**d**) Analysis of shrub FEve across forest types. Note: letters indicate significant differences in the one-way analysis of variance (one-way ANOVA).

Analysis of the functional diversity of shrubs in broadleaved and coniferous forests revealed that FDiv and functional evenness (FEve) were numerically higher in the coniferous forest, although these differences were not statistically significant (*p* > 0.05; Figure 3b,d). Similarly, FRic and Rao’s quadratic entropy (RaoQ) were lower in the broadleaved forest compared to the coniferous forest, but again, the differences were not significant (*p* > 0.05; Figure 3a,c). Although none of the functional diversity indices showed statistically significant differences, they consistently indicated a trend: compared to the broadleaved forest, the shrub community in the coniferous forest may be shaped by strong environmental filtering, resulting in an assemblage with a broader functional range, more even structural distribution, and a greater proportion of specialized species.

### 3.3. Shrub Biodiversity and Phylogenetic Diversity in Two Forest Types

**Figure 4:**
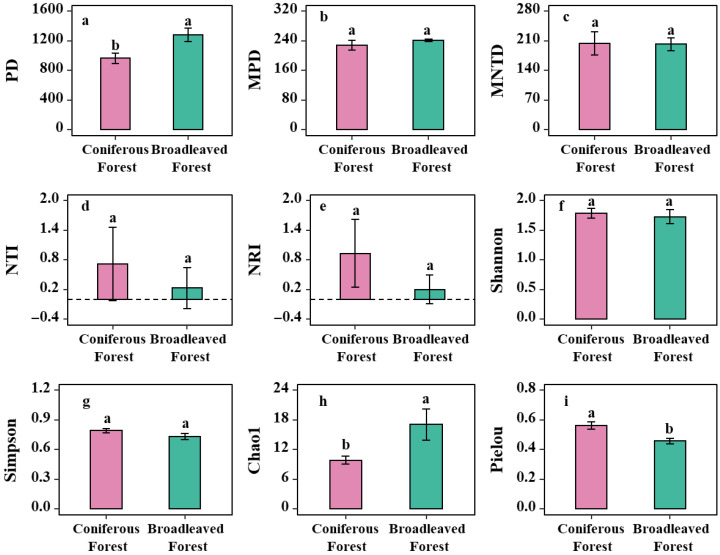
Differences in shrub phylogenetic diversity and biodiversity between two Forest types. **Note:** (**a**) Analysis of differences in shrub PD index across forest types; (**b**) Analysis of differences in shrub MPD index across forest types; (**c**) Analysis of differences in shrub MNTD index across forest types; (**d**) Analysis of differences in shrub NTI across forest types; (**e**) Analysis of differences in shrub NRI across forest types; (**f**) Analysis of differences in shrub Shannon-Wiener diversity index across forest types; (**g**) Analysis of differences in shrub Simpson’s diversity index across forest types; (**h**) Analysis of differences in shrub Chao1 index across forest types; (**i**) Analysis of differences in shrub Pielou’s evenness index across forest types. Note: Different letters indicate significant differences in the one-way analysis of variance (one-way ANOVA).

Analysis of shrub species diversity and phylogenetic diversity in the two forest types revealed that the phylogenetic diversity (PD) of shrubs in the coniferous forest was significantly lower than that in the broadleaved forest (*p* < 0.05; Figure 4a). Although the mean pairwise distance (MPD) and the mean nearest taxon distance (MNTD) of shrubs in the coniferous forest were slightly lower, on average, than those in the broadleaved forest, the differences were not statistically significant (*p* > 0.05; Figure 4b,c). These results indicate that the overall phylogenetic structure of the shrub communities is similar between the two forest types. Compared to the broadleaved forest, the shrub community in the coniferous forest exhibits greater selectivity from an evolutionary perspective, but this selective filtering has not led to a systematic change in the overall community phylogenetic structure.

Regarding the biodiversity indices, no significant differences were observed between the two forest types for the Shannon-Wiener index and the Simpson index (*p* > 0.05; Figure 4d,e). However, the Chao1 index was significantly higher in the broadleaved forest than in the coniferous forest (*p* < 0.05; Figure 4h), suggesting a richer potential species pool (i.e., a greater estimated total number of species) in the shrub layer of the broadleaved forest. This implies that the broadleaved forest environment, potentially characterized by more complex structure and diverse resources, can support the coexistence of a wider variety of shrub species. In contrast, the Pielou’s evenness index was significantly higher in the coniferous forest (*p* < 0.05; Figure 4i). This indicates a more uniform distribution of individuals among the different shrub species in the coniferous forest. Conversely, the lower evenness in the broadleaved forest suggests the presence of a few “dominant species” that account for a large proportion of individuals, while many other species are relatively rare. Therefore, the coniferous forest environment appears to exert a stronger environmental filter, which dominates the survival strategies and assembly of the shrub community therein.

### 3.4. Phylogenetic Structure of Shrub Communities

Both the NRI and the Nearest Taxon Index (NTI) were greater than zero in both forest types, with the mean values for both indices being higher in the coniferous forest than in the broadleaved forest (*p* > 0.05; Figure 4d,e). The fact that NRI > 0 across both forest types indicates that the overall phylogenetic structure of the shrub communities exhibits phylogenetic clustering. This means that the coexisting shrub species are more closely related phylogenetically than would be expected by random chance, suggesting that species with similar adaptive traits coexist. Similarly, NTI > 0 indicates that this phylogenetic clustering pattern also holds at the terminal tips of the phylogenetic tree (representing more recent evolutionary history), meaning that even closely related species within the community are phylogenetically clustered.

In summary, the fact that both NRI and NTI are greater than zero suggests that the shrub communities in both forest types exhibit a clustered phylogenetic pattern at both deep and recent evolutionary levels. This pattern is primarily driven by strong environmental filtering. The consistently lower values of both indices in the broadleaved forest compared to the coniferous forest suggest that the environmental filtering is likely stronger in the coniferous forest. The environmental conditions in the coniferous forest are arguably more stringent and specific, acting as a stronger filter that allows only species from specific evolutionary lineages with particular adaptations to survive, resulting in a community where the constituent species are more closely related.

Although the differences in NTI and NRI between the two forest types were not statistically significant (NTI: t = −0.86853, *p* = 0.4065; NRI: *p* = 0.349), the analysis of effect sizes provides further insight. For NTI, Cohen’s dwas 0.33 (95% CI: −0.82, 1.46) and Hedges’ gwas 0.31 (95% CI: −0.75, 1.35), indicating a small to medium effect size for the difference between forest types. Similarly, for NRI, Cohen’s dwas 0.57 (95% CI: −0.60, 1.71) and Hedges’ gwas 0.52 (95% CI: −0.56, 1.58), also suggesting a small to medium practical difference in the phylogenetic structure of the shrub communities between the two forest types.

### 3.5. Analysis of Differences in Assembly Mechanisms Between the Two Shrub Types

**Figure 5:**
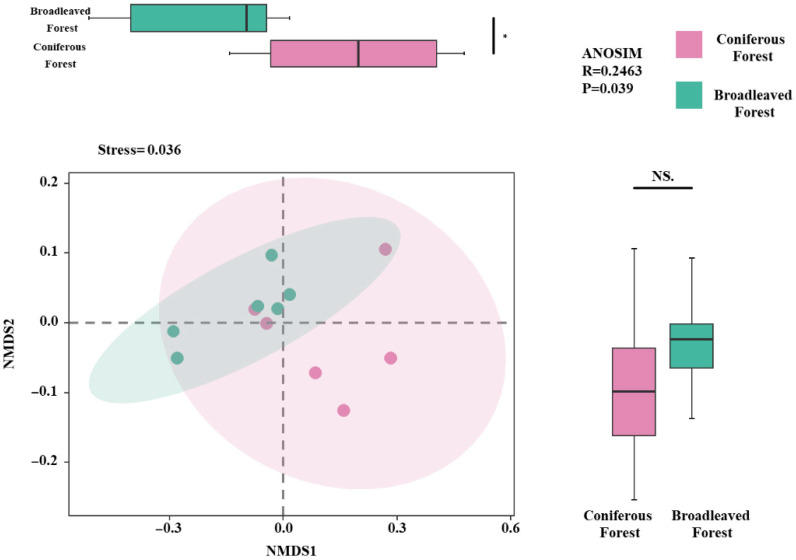
Non-metric Multidimensional Scaling (NMDS) analysis under two forest types. Note: * denotes *p* < 0.05. NMDS plot showing shrub community composition across sampling plots, with ellipses indicating 95% confidence intervals and colors representing different forest types. NS—Not Significant.

Non-metric multidimensional scaling (NMDS) was used to analyze the phylogenetic diversity, functional diversity, and species diversity of the shrubs. The results indicated that the shrub communities in the two forest types exhibited significant differences primarily along the NMDS1 axis (*p* < 0.05; Figure 5), while no significant difference was observed along the NMDS2 axis. The stress value of the NMDS model was 0.036, indicating a good fit and high reliability of the ordination results in representing the actual dissimilarities among communities.

Furthermore, an analysis of similarities (ANOSIM) test was conducted to verify the significance of the differences between the two forest types. The results showed a significant distinction between the shrub communities of the two forest types (R = 0.2463, *p* = 0.036). The positive R-value indicates that the differences between groups (inter-forest type differences) are greater than the differences within groups (intra-forest type variations), and this difference is statistically significant.

### 3.6. Influence of Environmental Factors on Understory Shrubs

**Figure 6:**
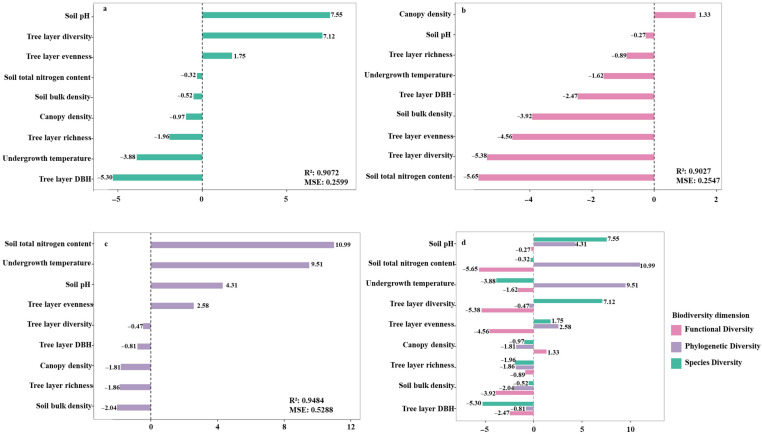
Random Forest Analysis of the Impact of Environmental Factors and Tree Layer Factors on Shrub Community Assembly. Note: (**a**) Importance of environmental factors on shrub species diversity; (**b**) Importance of environmental factors on shrub functional diversity; (**c**) Importance of environmental factors on shrub phylogenetic diversity; (**d**) Importance of environmental factors on shrub community assembly mechanisms.

To investigate the effects of abiotic (environmental) and biotic (tree layer) factors on the assembly mechanisms of shrub communities, this study analyzed the shrub communities from three dimensions—species diversity, phylogenetic diversity, and functional diversity—evaluating the respective influence of environmental and tree layer factors. Random forest analysis revealed that soil bulk density, soil pH, and tree layer diversity positively influenced shrub species diversity. In contrast, soil total nitrogen content, tree layer richness, canopy density, understory temperature, tree layer evenness, and tree-layer basal area exhibited negative effects. Among these, soil pH was the most important positive predictor of shrub diversity, while tree-layer basal area showed the strongest negative effect (Figure 6a; R^2^ = 0.9072, MSE = 0.2599). For shrub functional diversity, canopy density had a positive influence, whereas soil total nitrogen content and tree layer diversity exerted negative effects (Figure 6b; R^2^ = 0.9027, MSE = 0.2547). Regarding shrub phylogenetic diversity, environmental factors including soil total nitrogen content, understory temperature, and soil bulk density, along with tree layer evenness, showed positive driving effects. Conversely, tree layer diversity, tree layer richness, canopy density, and tree layer DBH were negatively correlated. Specifically, soil total nitrogen and understory temperature had relatively strong positive effects, while canopy density exhibited a substantial negative effect (Figure 6c; R^2^ = 0.9484, MSE = 0.5288). Overall, factors such as tree layer richness, soil bulk density, and tree layer DBH consistently showed negative driving effects across the three functional modules of shrubs, indicating a concentrated influence. The effects of other factors were more dispersed (Figure 6d). These findings align with studies in other regions, which also found tree layer structure (particularly basal area) to be a key factor influencing understory shrubs, sometimes negatively, and soil properties like pH to be significant drivers. The results underscore that the assembly of shrub community structure is influenced by a complex interplay of abiotic environmental filtering and biotic interactions from the tree layer, with competition exclusion playing a major role. Among these, tree layer structure—especially basal area—is the most critical negative factor constraining shrub diversity, while soil pH is the most critical positive factor sustaining it.

### 3.7. Redundancy Analysis (RDA) of Factors Influencing Shrub Functional Diversity

**Figure 7:**
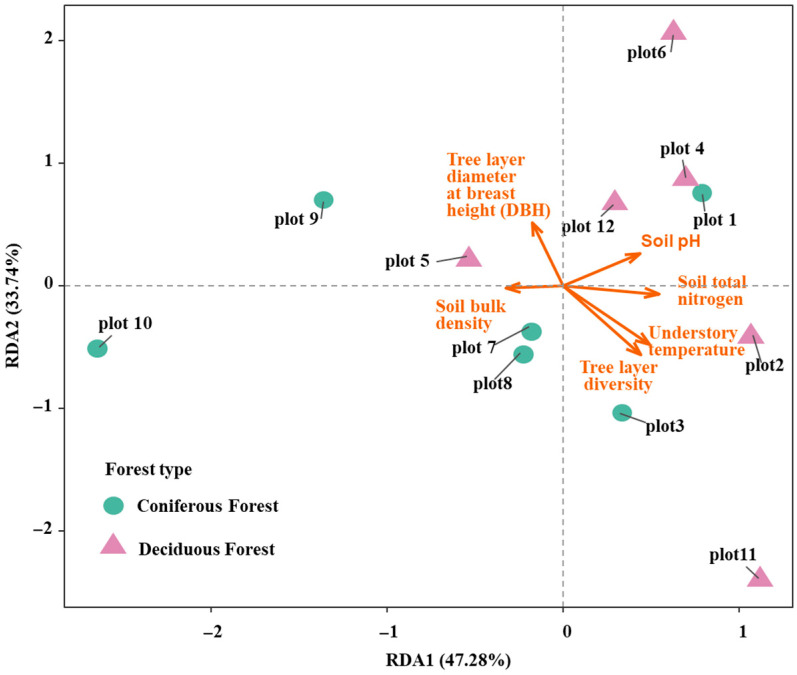
RDA of shrub functional diversity and environmental variables under two forest types.

To assess the combined effects of tree layer factors (biotic factors) and environmental factors (abiotic factors) on shrub functional diversity, a redundancy analysis (RDA) was conducted. The RDA model explained a total of 80.75% of the variation in shrub functional diversity. Specifically, RDA axis 1 accounted for 47.28% of the variance, while RDA axis 2 explained 33.74% (Figure 7). The overall model was statistically significant and demonstrated a good fit (R^2^ = 0.29, *p* = 0.042), indicating its robustness in explaining the observed patterns. Within the model, soil total nitrogen content and soil pH exhibited statistically significant effects (*p* < 0.05). The results showed a negative correlation between soil bulk density and soil pH. In terms of contributions to the RDA axes, tree diameter at breast height (DBH) and tree layer diversity contributed substantially to RDA axis 2. Conversely, soil bulk density and soil total nitrogen had strong loadings on RDA axis 1, though they were negatively correlated with each other. These findings suggest that the assembly of shrub communities and their functional diversity are driven by distinct processes in the two forest types. In the broadleaved forest, a more complex process predominates, co-regulated by a combination of soil factors and multiple environmental variables, with its development primarily constrained by external resources. In contrast, in the coniferous forest, tree DBH (indicating biotic competition and tree layer structure) and soil bulk density play more critical roles, shaping a community assembly process dominated by strong environmental filtering, which is consistent with the results from the Random Forest analysis.

## 4. Discussion

### 4.1. Differentiation in Understory Shrub Functional Traits Reflects Distinct Ecological Strategies

Plant functional traits, whether examined individually or in combination, serve as indicators of adaptation and response to specific environmental conditions. These traits elucidate the trade-offs plants make in resource acquisition and utilization, further revealing the distinct life–history strategies employed by plants across different habitats [40]. The findings of this study demonstrate significant differences in the leaf functional traits of understory shrubs between broadleaved and coniferous forests. The shrub community in the broadleaved forest exhibits a trait syndrome characterized by lower Specific Leaf Area (SLA), lower chlorophyll content, and higher Leaf Dry Matter Content (LDMC), indicating a resource-conservative strategy [41]. Such a combination of traits can reduce niche overlap and competition, thereby promoting biodiversity and enhancing ecosystem stability [42].

In forests characterized by resource constraints, such as those under stronger environmental filtering, understory shrubs allocate more resources to withstand stressful conditions. Consequently, they adopt a slow-return, resource-saving strategy, developing a suite of traits including higher LDMC, lower SLA, and lower chlorophyll content. This trait spectrum facilitates greater nutrient storage and slower growth rates [43]. The significant difference in the Chao1 index further corroborates the divergence in survival strategies of shrubs between the two forest types, suggesting a richer potential species pool in the broadleaved forest environment.

### 4.2. Functional Diversity and Phylogenetic Structure Reveal Distinct Community Assembly Mechanisms

The Net Relatedness Index (NRI) and Nearest Taxon Index (NTI) are essential metrics for elucidating ecological processes underlying community assembly, such as environmental filtering, competitive exclusion, and stochastic processes [44]. The integration of functional diversity and phylogenetic structure provides robust evidence for deciphering these mechanisms. Both coniferous and broadleaved forests exhibited mean NRI and NTI values greater than zero, indicating a phylogenetically clustered structure consistent with strong environmental filtering that selects for closely related species with similar adaptive traits [45,46]. Although both forest types displayed phylogenetic clustering, the significantly higher NRI and NTI values in the coniferous forest reflect a more intense environmental filtering process. The harsher understory conditions of the coniferous forest—characterized by lower light availability, acidic soils, and potential allelopathic effects—have shaped a shrub community with more recent evolutionary divergence and closer phylogenetic relatedness. While the overall phylogenetic structure is qualitatively similar between forest types, the strength of the underlying ecological process—environmental filtering—differs substantially [36]. Furthermore, the functional evenness observed in the coniferous forest suggests that distantly related species may achieve coexistence through functional divergence and niche differentiation. By increasing functional differences and reducing niche overlap, these species distribute more evenly in functional space, thereby mitigating interspecific competition and promoting stable coexistence [47]. Notably, the NTI values for shrubs in both forest types fell within the range of −1.96 to 1.96, which overlaps with the confidence interval expected under a random model. This indicates that stochastic processes may also play a non-negligible role in structuring the understory communities, as has been demonstrated in other ecosystems [48]. Thus, the assembly of shrub communities in both forest types is likely governed by a combination of deterministic (e.g., environmental filtering) and stochastic processes, with their relative importance mediated by forest-type-specific conditions.

### 4.3. Key Environmental Drivers of Functional Trait Patterns

Environmental heterogeneity is a primary cause of divergence in plant functional traits among vegetation types [49]. As a critical environmental factor, soil plays a significant role in structuring understory plant communities [50]. Research has consistently shown that soil nutrient content is a key determinant of species diversity. The present study, based on Random Forest analysis and Redundancy Analysis (RDA), aligns with previous findings in demonstrating that soil factors exert a strong influence on shrub phylogenetic diversity, with soil pH and soil total nitrogen being particularly important. Soil pH can directly or indirectly impact understory biodiversity. Some studies suggest a hump-shaped relationship between plant diversity and soil pH, where diversity initially increases with rising pH, peaks at an optimal value, and then declines as pH increases further [51]. Under the present experimental conditions, the results did not significantly align with those previously reported, which may be attributable to regional specificity. Simultaneously, soil total nitrogen (N) showed high importance for phylogenetic diversity in our study. Generally, phylogenetic diversity is thought to increase with higher soil total N, as nutrient-rich conditions may support a wider range of lineages [52]. While this study provides insights into the key environmental drivers of shrub layer functional diversity, several limitations should be acknowledged. The research relied on plot-level data from a specific regional forest ecosystem, which may limit the direct extrapolation of findings to other biogeographic contexts or broader spatial scales.

The tree layer, as a dominant biotic factor in forest ecosystems, exerts a substantial and complex influence on understory plants. Our results indicate that tree layer factors, particularly basal area, generally show significant negative effects on shrub layers (Figure 5). This is likely due to intense resource competition, where trees suppress shrub development by limiting light availability and soil resources [53]. However, an interesting finding from our study is that tree layer evenness and diversity exhibited positive effects on shrub biodiversity. This positive effect could be attributed to a “big-tree effect” or, more precisely, to the habitat complexity created by a multi-layered canopy [54]. A more complex vertical structure may promote niche differentiation, allowing for a greater number of plant species to coexist by enabling more efficient capture and utilization of light resources, thereby helping to maintain higher species diversity [55]. In summary, the functional trait patterns of understory shrubs are co-driven by both abiotic soil factors (e.g., pH, total nitrogen) and biotic tree layer factors. The interaction between resource competition (often manifested as negative effects from dominant trees) and facilitation through habitat complexity (positive effects from a diverse tree layer) collectively shapes the assembly and diversity of shrub communities.

## 5. Conclusions

This study reveals fundamental differences in the assembly mechanisms of shrub communities beneath coniferous and broadleaved forests. Coniferous forests, through strong environmental filtering, form communities with fewer species but more even distribution, phylogenetic clustering, and functional specialization. In contrast, broadleaved forests maintain higher species richness, yet their communities are often dominated by a few superior species and exhibit higher functional redundancy. Analyses indicate that the tree-layer basal area is a key negative factor suppressing shrub diversity, while soil pH serves as an important positive driver. Future research could integrate long-term monitoring with experimental manipulation to deeply analyze the combined effects of multiple environmental factors and biological interactions on community dynamics, thereby providing a more precise ecological basis for forest management and biodiversity conservation. However, this study has certain limitations. It did not investigate other metallic elements or additional environmental variables. Future research should include a wider range of factors to more systematically elucidate the underlying mechanisms. Additionally, the limited sample size prevented the development of a comprehensive model to examine how tree-layer factors interact with environmental variables in shaping shrub-layer community assembly.

## Figures and Tables

**Table 1:** Comparison of Observed Environmental Factors Between the Two Forest Types.

Type	Plot	Soil Total Nitrogen Content/(mg/kg)	Soil pH	Canopy Density/%	Soil Bulk Density (g/cm^3^)	Undergrowth Temperature/°C
A	plot1	3641.19	4.60	75.50	1.50	30.42
B	plot2	3307.12	4.90	80.00	1.27	30.42
A	plot3	4195.45	4.80	81.50	1.36	29.09
B	plot4	1649.52	5.50	85.00	1.10	28.05
B	plot5	998.97	5.30	96.00	1.06	28.05
B	plot6	1925.94	5.40	92.00	1.10	28.05
A	plot7	1164.56	4.90	86.00	1.34	30.42
A	plot8	2023.29	4.30	84.00	1.28	30.42
A	plot9	840.08	4.40	78.50	1.43	27.65
A	plot10	511.07	4.40	83.25	1.39	27.65
B	plot11	1716.92	5.10	97.00	1.06	32.25
B	plot12	2268.33	5.40	96.00	1.13	31.00

Note: A denotes the coniferous forest; B denotes the broadleaved forest.

**Table 2:** Investigation of Arbor Layer Structure.

Tepy	Plot	Tree Layer DBH/mm	Tree Layer Diversity	Tree Layer Richness	Tree Layer Evenness
A	plot1	209.89	0.62	2	0.90
B	plot2	224.14	1.01	4	0.73
A	plot3	165.15	0.97	4	0.70
B	plot4	200.44	0.59	3	0.53
B	plot5	201.24	0.88	3	0.80
B	plot6	300.70	0.26	2	0.37
A	plot7	200.10	0.97	3	0.89
A	plot8	269.45	0.83	3	0.75
A	plot9	245.54	0.69	4	0.50
A	plot10	218.10	0.14	2	0.21
B	plot11	146.61	1.36	6	0.76
B	plot12	163.72	0.96	4	0.69

Note: A denotes the coniferous forest; B denotes the broadleaved forest.

**Table 3:** Shrub Community Composition Survey.

Family	Genus	Species	Coniferous Forest Relative Abundance	Broadleaf Forest Relative Abundance
Aquifoliaceae	*Ilex*	*Ilex aculeolata*	13.15	1.42
		*Ilex cornuta*	0.17	0.31
Berberidaceae	*Nandina*	*Nandina domestica*	–	1.29
Caprifoliaceae	*Symphoricarpos*	*Symphoricarpos orbiculatus*	–	0.06
	*Lonicera*	*Lonicera japonica*	–	0.12
Cornaceae	*Cornus*	*Cornus alba*	–	0.06
Elaeagnaceae	*Elaeagnus*	*Elaeagnus umbellata*	–	0.06
Ericaceae	*Lyonia*	*Lyonia ovalifolia*	0.69	0.18
Hamamelidaceae	*Fortunearia*	*Fortunearia sinensis*	–	0.12
Lamiaceae	*Clerodendrum*	*Clerodendrum cyrtophyllum*	9.95	0.12
Mallotus	*Mallotus*	*Mallotus repandus*	–	5.17
Moraceae	*Broussonetia*	*Broussonetia kaempferi*	1.73	14.77
	*Ficus*	*Ficus pandurata*	–	7.08
Myricaceae	*Myrica*	*Myrica cerifera*	–	0.06
Myrtaceae	*Syzygium*	*Syzygium buxifolium*	1.38	0.18
Oleaceae	*Ligustrum*	*Ligustrum sinense*	0.09	0.00
Phyllanthaceae	*Phyllanthus*	*Phyllanthus glaucus*	1.73	8.62
	*Glochidion*	*Glochidion puberum*	–	0.06
	*Pittosporum*	*Pittosporum tobira*	0.61	7.32
Platanaceae	*Platanus*	*Platanus acerifolia*	0.35	0.06
Primulaceae	*Myrsine*	*Myrsine africana*	0.43	0.06
Ranunculaceae	*Clematis*	*Clematis terniflora*	–	0.06
Rhamnaceae	*Sageretia*	*Sageretia thea*	0.52	3.26
Rosaceae	*Rubus*	*Rubus corchorifolius*	7.01	1.17
	*Rhaphiolepis*	*Rhaphiolepis indica*	4.93	1.23
	*Rosa*	*Rosa multiflora*	–	13.17
		*Rosa laevigata*	6.06	2.46
Rubiaceae	*Gardenia*	*Gardenia jasminoides*	23.62	11.75
	*Paederia*	*Paederia foetida*	–	10.77
	*Serissa*	*Serissa japonica*	2.42	2.95
Rutaceae	*Zanthoxylum*	*Zanthoxylum armatum*	–	0.06
Salicaceae	*Xylosma*	*Xylosma congesta*	–	0.18
Smilacaceae	*Smilax*	*Smilax china*	12.11	1.11
Symplocaceae	*Symplocos*	*Symplocos tanakana*	11.42	2.34
		*Symplocos lancifolia*	0.09	–
Theaceae	*Camellia*	*Camellia japonica*	1.56	0.55
Verbenaceae	*Vitex*	*Vitex negundo* var. *cannabifolia*	–	0.62
Vitaceae	*Causonis*	*Causonis japonica*	–	0.18
	*Parthenocissus*	*Parthenocissus quinquefolia*	–	0.92
	*Ampelopsis*	*Ampelopsis glandulosa*	–	0.06

## Data Availability

Data can be made available on reasonable request.
